# MFP1 defines the subchloroplast location of starch granule initiation

**DOI:** 10.1073/pnas.2309666121

**Published:** 2024-01-08

**Authors:** Mayank Sharma, Melanie R. Abt, Simona Eicke, Theresa E. Ilse, Chun Liu, Miriam S. Lucas, Barbara Pfister, Samuel C. Zeeman

**Affiliations:** ^a^Institute of Molecular Plant Biology, Department of Biology, ETH Zurich, 8092 Zurich, Switzerland; ^b^Scientific Center for Optical and Electron Microscopy, ETH Zurich, 8093 Zurich, Switzerland

**Keywords:** plant metabolism, starch biosynthesis, starch granule initiation, coiled-coil protein, chloroplast

## Abstract

Starch is one of the major carbohydrate storage compounds in plants. The biogenesis of starch granules starts with the formation of initials, which subsequently expand into granules. Several coiled-coil domain-containing proteins have been previously implicated with the initiation process, but the mechanisms by which they act remain largely elusive. Here, we demonstrate that one of these proteins, the thylakoid-associated MAR-BINDING FILAMENT-LIKE PROTEIN 1 (MFP1), specifically determines the subchloroplast location of initial formation. The expression of MFP1 variants “mis”-targeted to specific locations within chloroplasts in Arabidopsis results in distinctive shifts in not only how many but also where starch granules are formed. Importantly, “re” localizing MFP1 to the stromal face of the chloroplast’s inner envelope is sufficient to generate starch granules in this aberrant position. These findings provide compelling evidence that a single protein MFP1 possesses the capacity to direct the initiation and biosynthesis machinery of starch granules.

## Significance

In leaf chloroplasts, starch granules are recurrently synthesized in the stromal space between the thylakoid membranes. Here, we provide unambiguous evidence that the thylakoid-associated protein MAR-BINDING FILAMENT-LIKE PROTEIN 1 (MFP1) determines this location of starch granule biogenesis within the chloroplast. Together with its interacting protein partners, MFP1 can initiate starch granules at a specific subchloroplast location given that it is anchored to a membrane compartment. This study establishes the first step of starch granule initiation and significantly advances our understanding of starch biosynthetic machinery.

Starch is a vital plant polymer, not only because of its omnipresence in human nutrition but also as an industrial feedstock. Starch consists of semicrystalline granules that contain two different glucose polymers: amylopectin and amylose. Both polymers consist of covalently linked glucose units but differ in their structural complexity; while amylose molecules are essentially linear α-1,4 linked chains, amylopectin is a larger polymer, hierarchically branched via α-1,6 glycosidic linkages. The branching pattern of amylopectin allows secondary and tertiary structures to be adopted that ultimately result in the crystallization of parts of the molecule, hence the semicrystalline nature of starch granules. Starch is produced in a variety of plant tissues and often categorized as two major types: storage starch and transient starch. Storage starch is typically synthesized within the amyloplasts of nongreen heterotrophic tissues, while transient starch forms on a daily basis inside the chloroplasts of green, photosynthetically active tissues. We recently demonstrated that the biosynthesis of transient starch granules in Arabidopsis leaves begins with the assembly of multiple smaller structures—the so-called starch initials—that coalesce with one another and subsequently grow anisotropically into lenticular, semicrystalline granules (1).

While the enzymatic mechanisms of starch polymer biosynthesis have been thoroughly studied (2), the early processes that create starch initials and how granule expansion is controlled are not well understood. Neither the precise nature or origin of the glucan substrates is known, nor have the mechanisms for assembling and/or elaborating them into a suitable substrate for the starch biosynthetic enzymes been conclusively elucidated. However, several protein factors influencing these early processes have been identified. The first is a member of the starch synthase family of enzymes: STARCH SYNTHASE 4 (SS4). Arabidopsis mutants lacking SS4 not only show a marked reduction in the number of starch granules forming per chloroplast, but these granules are also near-spherical instead of lenticular (3). Interestingly, these two effects were found to be separately conditioned by different parts of the SS4 protein; while the catalytic C-terminal part alone is sufficient to regulate granule numbers, the nonenzymatic N terminus is required to mediate the establishment of a lenticular granule morphology (4). More recently, several proteins lacking enzymatic activity were found to play a part, with the respective mutants producing fewer starch granules per chloroplast, albeit with a wild-type (WT)-like granule morphology. These proteins include a noncanonical, nonenzymatic starch synthase (STARCH SYNTHASE 5) (5), two members of the PTST (PROTEIN TARGETING TO STARCH) protein family (PTST2 and PTST3) (6), and two large coiled-coil domain-containing proteins named MRC/PII1 (MYOSIN-RESEMBLING CHLOROPLAST PROTEIN or PROTEIN INVOLVED IN INITIATION 1) (7, 8) and MFP1 (MAR-BINDING FILAMENT-LIKE PROTEIN 1) (7). These proteins are collectively termed starch granule initiation proteins.

A prominent feature of the starch granule initiation proteins is that they engage in extensive and likely versatile protein–protein interactions. PTST2 physically interacts with SS4, and MFP1 and MRC were originally identified in immunoprecipitation experiments and yeast-2-hybrid assays via interactions with PTST2 and/or SS4 (7, 8). In addition to SS4, MRC was further shown to bind to SS5, suggesting that some of these interactions may be multivalent or mutually exclusive (5). While the interaction between PTST2 and SS4 has been interpreted such that the former supplies the latter with selected oligosaccharides for elaboration (6), the interaction of MFP1 and PTST2 likely influences where this process happens at the subchloroplast level. Among the initiation proteins, MFP1 is unique in that it is firmly attached to the chloroplast thylakoid membranes. While in WT plants, PTST2 is partially associated with the thylakoids, too, its localization changes in the absence of MFP1, with the protein then residing completely in the chloroplast stroma (7). Thus, the subchloroplast localization of PTST2 is dependent on MFP1.

**Fig. 1.**
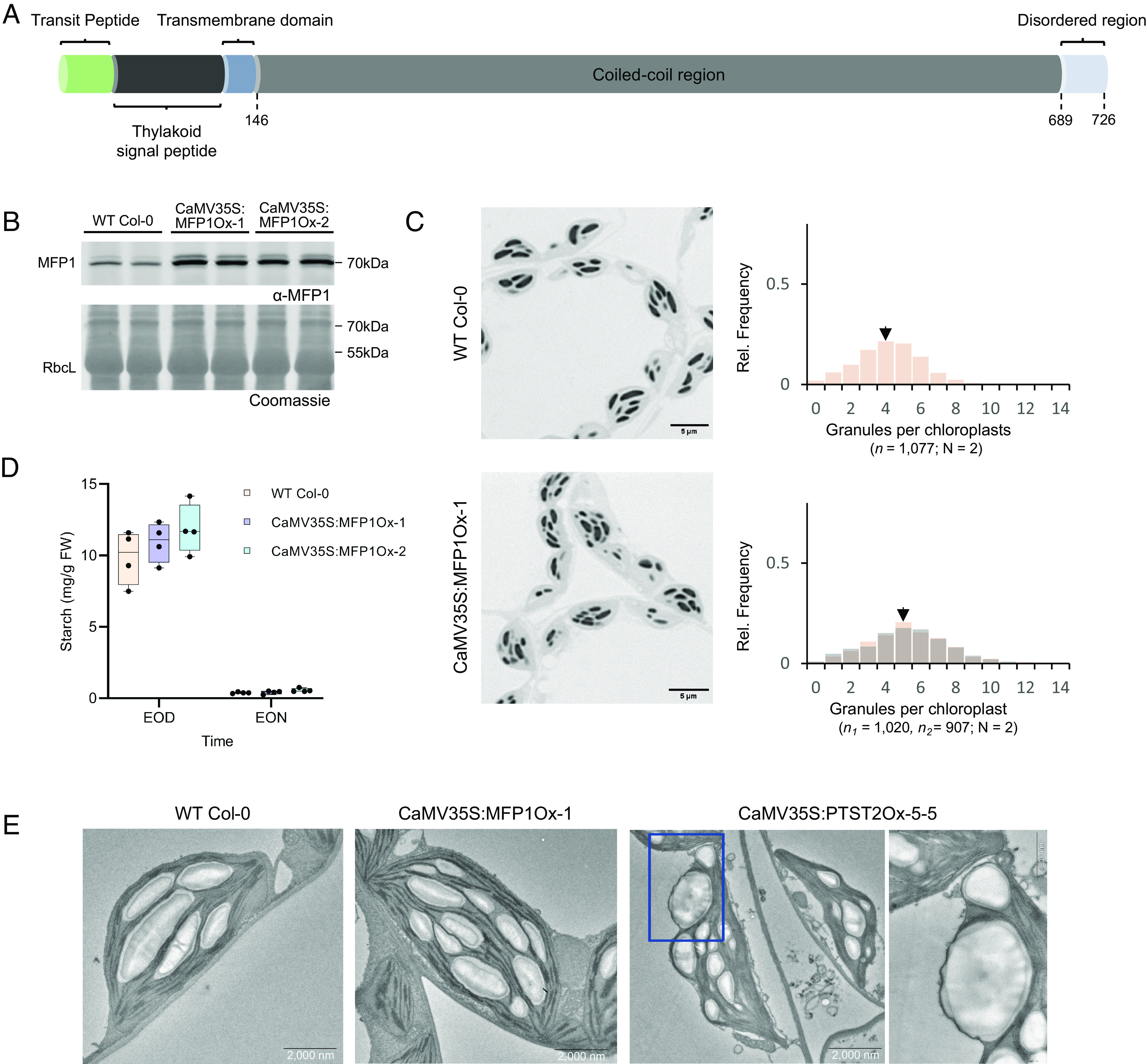
MFP1 overexpression affects the numbers of starch granules forming in chloroplasts. (*A*) Schematic representation of the MFP1 protein. (*B*) Overexpression of MFP1 in the two selected lines, confirmed by immunoblotting using antibodies raised against recombinant MFP1 protein. Gels were loaded on an equal fresh weight basis; two biological replicate plants were sampled for each line. The Coomassie-stained membrane shows equal total protein loading per sample. (*C*) LM images of toluidine blue stained semithin sections obtained from fixed and embedded leaf tissues harvested at the EOD. Starch granules are visible as dark structures inside the chloroplasts. Manual counts of starch granules per chloroplast section (n) are displayed as histograms (*Righthand*). Each histogram represents starch granule counts from at least two biological replicates (N) per genotype, and the resulting histograms of two independent transgenic lines are overlaid for the CaMV35S:MFP1Ox. Black arrows indicate the median. On the *Y* axis is the relative frequency of occurrence of the specified number of granules per chloroplast (*X* axis). (*D*) Starch quantification of MFP1 overexpresser lines. Entire rosettes (n = 4 biological replicates) were harvested at the EOD or EON, the starch therein extracted and measured using an enzymatic assay after digestion to glucose. An unpaired *t* test was performed for statistical analysis, and the difference was not significant compared to WT (Col-0) samples. (*E*) TEM images of representative chloroplasts in leaves of the indicated lines harvested at the EOD.

The N-terminal part of MFP1 is predicted to comprise a plastid-specific transit peptide, but also a thylakoid twin-arginine-translocation (Tat) signal sequence, and a short transmembrane domain, while the C-terminal part consists of long coiled-coil motifs (Fig. 1*A*) (9). Tat transport is one of the pathways that facilitate protein import/integration into the thylakoids (10), and it was hypothesized that MFP1 becomes anchored there with its C-terminal coiled-coil domain facing into the stroma, allowing the conglomeration of other granule initiation proteins (11). Here, we sought to test this hypothesized function of MFP1 by creating and characterizing Arabidopsis lines expressing MFP1 variants that were purposefully “retargeted” to distinct chloroplast subcompartments. This approach allowed us to reveal the crucial role of MFP1 transmembrane anchorage and thylakoid localization in the process of starch granule initiation.

## Results

### MFP1 Overexpression Increases the Number of Starch Granules Per Chloroplasts.

It was previously reported that Arabidopsis mutants lacking MFP1 produce fewer but larger starch granules per chloroplast than the WT, even though the overall starch contents were comparable (7). To assess whether MFP1 limits the number of starch granules, we overexpressed Arabidopsis MFP1 using the Cauliflower Mosaic Virus (CaMV) 35S promoter in WT Arabidopsis plants. Two independent transgenic lines (CaMV35S:MFP1Ox), each overexpressing MFP1 (as confirmed by immunoblotting; Fig. 1*B*), were selected for detailed phenotypic characterization. We observed a shift toward slightly increased numbers in the lines overexpressing MFP1 as compared to the WT (Fig. 1*C*). Leaf starch quantifications at the end of day (EOD) and end of night (EON) suggested that MFP1 overexpression has no or little effect on total starch content at either time point (Fig. 1*D*).

We next extracted the starch granules from leaf tissue harvested at the EOD and analyzed their sizes and morphologies via scanning electron microscopy (SEM) and flow cytometry (FC) (*SI Appendix*, Fig. S1) (12). The FC profiles and electron micrographs indicate a subtle shift toward smaller size in lines overexpressing MFP1 while overall starch granule shape remained unaffected. The production of increased numbers of starch granules was previously observed in Arabidopsis lines overexpressing PTST2, an interaction partner of MFP1 (6). However, in that case, the effect on granule numbers and morphology was stronger; the granules were smaller and displayed morphological abnormalities, being more spherical than lenticular.

We further compared the two overexpression lines’ phenotypes by assessing the appearance and distribution of their starch granules using transmission electron microscopy (TEM). Starch granules have been previously shown to form in dedicated stromal spaces or “pockets” between thylakoid membranes (1). Indeed, the granules in WT chloroplasts were exclusively observed in such spaces (Fig. 1*E*). The granules in MFP1Ox chloroplasts, despite being more numerous than in the WT, also resided in stromal spaces surrounded by thylakoid membranes. Interestingly however, the numerous granules in the PTST2Ox line often appeared to be distributed more randomly in the chloroplast (6), with some localized directly adjacent to the chloroplast envelope.

Compared to the numerous, variably sized granules in the PTST2Ox line (6), the increase in the number of starch granules per chloroplast was limited in the case of MFP1Ox. This limitation may be attributed to the restricted availability of PTST2 and, consequently, other components of the initiation machinery. To test this, we generated lines that overexpressed both MFP1, driven by the CaMV35S promoter, and the PTST2 gene, driven by the Ubiquitin 10 promoter. The use of two different promoters aimed to prevent potential transcriptional silencing. We isolated two independent lines and analyzed leaf starch granule numbers and morphology. Interestingly, we observed a significant enhancement in the number of starch granules per chloroplast, with several chloroplasts now harboring more than 20 starch granules (*SI Appendix*, Fig. S2). Subsequent examination using TEM revealed that the granules were located in their typical positions, between the thylakoid membranes. These microscopy images, combined with FC analysis of extracted starch, showed that while the granule size was reduced, the granule morphology remained similar to that of the WT and MFPOx lines. (*SI Appendix*, Fig. S3). However, it is important to note that the expression levels of PTST2 in these double overexpresser lines were not as high as in the single PTST2Ox line. It remains possible that higher levels of PTST2 overexpression could still cause ectopic granule initiation, even in the presence of elevated MFP1 levels. Collectively, these data suggest an important mechanistic difference in the way starch granule numbers are increased by overexpressing the two proteins.

### MFP1 Is Anchored to the Thylakoid Membranes via Its N-terminal Transmembrane Domain.

**Fig. 2.**
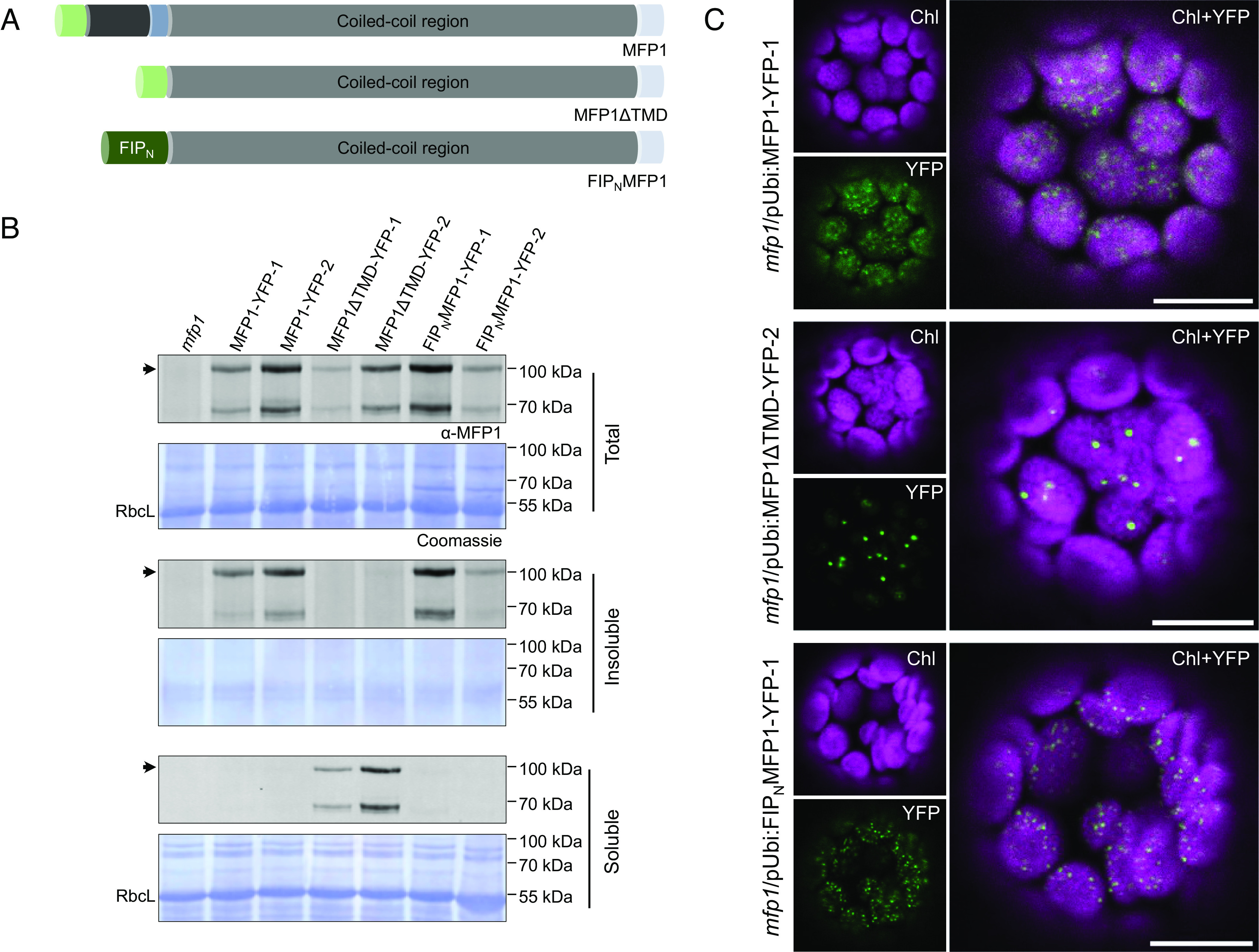
Truncated or chimeric MFP1 variants display characteristic subchloroplast distribution pattern. (*A*) Schematic representation of the MFP1 variants constructed (*B*) Immunoblots depicting the localization of various YFP-tagged MFP1 variants (expressed in the *mfp1* mutant background) in total, membrane-bound, and soluble protein fractions. Upper bands represent the YFP-tagged MFP1 variant and the lower bands are possibly the degradation products. Two independent transgenic lines are analyzed per protein variant. The Coomassie-stained membrane shows equal protein loading per sample and the presence/absence of the soluble chloroplast protein, large subunit of Rubisco (RbcL). (*C*) Subchloroplast localization pattern of the expressed YFP-tagged MFP1 variants, as assessed by confocal microscopy. Mesophyll chloroplasts were imaged in protoplasts isolated from leaf tissues of transgenic lines. Images are presented as chlorophyll channel (false color magenta), YFP channel (false color green), or the overlay of both channels (*Right*). (Scale bar, 10 µm.)

In addition to its chloroplast transit peptide, the MFP1 N terminus features a highly conserved thylakoid twin-arginine translocase (Tat) signal peptide and a small hydrophobic region (Fig. 1*A* and *SI Appendix*, Fig. S4). Proteins with similar characteristics, such as Pftf and Ftsh2, have been previously shown to interact with the thylakoid Tat subunits, ultimately resulting in their release into the membrane with their C-terminal parts facing the plastid stroma (13, 14). It is thus likely that MFP1 is similarly inserted, with its C-terminal coiled-coil region exposed into the stroma. To assess the importance of MFP1’s thylakoid signal peptide and transmembrane domain, we created transgenic Arabidopsis lines expressing an MFP1 variant devoid of both (hereafter referred to as MFP1ΔTMD). We also created lines expressing a chimeric protein carrying the N-terminal transit peptide and transmembrane domain (N1-82) of a thylakoid-associated protein FTSH5 INTERACTING PROTEIN 1 (FIP1) fused to the C-terminal coiled-coil region of MFP1 (hereafter referred to as FIP_N_MFP1) (Fig. 2*A*). FIP1 is a recently characterized thylakoid membrane protein that carries a transmembrane domain but no Tat-specific signal sequence (15). Both MFP1 variants, as well as WT MFP1, were expressed in the *mfp1* mutant background under the control of the ubiquitin promoter and fused to a C-terminal YFP (yellow fluorescent protein) tag. After confirming expression of the protein variants by immunoblotting, two independent transgenic lines were selected of each for further analysis. We first determined the subchloroplast localization of the expressed MFP1 variants extracting total, soluble, and membrane-bound protein fractions from leaves of the different lines and analyzing them via immunoblotting (Fig. 2*B*). The fractionation of total leaf protein in soluble and insoluble fractions yields comparable pattern of MFP1 and PTST2 distribution to the fractionation of chloroplasts in stroma and thylakoids (7). As expected, MFP1ΔTMD-YFP was detectable in the soluble fraction, while both MFP1-YFP and FIP_N_MFP1-YFP were found in the membrane fraction. This confirms that the presence of a signal peptide and transmembrane domain is essential to the thylakoid membrane association of MFP1.

MFP1 was reported to localize to punctate structures within the chloroplast (7). To determine whether this distribution pattern is conditioned by the protein’s thylakoid anchorage, we used confocal microscopy to examine the in-vivo localization of the MFP1ΔTMD-YFP and FIP_N_MFP1-YFP protein variants in both epidermal leaf cells and isolated mesophyll protoplasts of the transgenic Arabidopsis lines. Consistent with earlier results, the line expressing WT MFP1-YFP formed punctate fluorescent structures within the chloroplasts of both cell types. Surprisingly, puncta were still present, but much less numerous in the lines expressing the soluble MFP1ΔTMD-YFP protein (Fig. 2*C* and *SI Appendix*, Fig. S5*A*). The attachment of the FIP N terminus, in case of the FIP_N_MFP1-YFP protein, restored the localization pattern with respect to the number of puncta in mesophyll cell chloroplasts (Fig. 2*C*), although fewer were seen in epidermal cell chloroplasts (*SI Appendix*, Fig. S5*A*). Nevertheless, they were more frequent and homogeneously distributed compared with those in the MFP1ΔTMD-YFP lines.

To further dissect the influence of MFP1’s coiled-coil domain on its localization pattern, we fused the N-terminal region of FIP directly to YFP (FIP_N_-YFP) and compared its localization with FIP_N_MFP1-YFP in *N. benthamiana*. The FIP thylakoid anchor alone conferred a punctate YFP distribution pattern, suggesting homogeneous distribution of the fusion protein at the thylakoid membranes. However, the presence of the MFP1 coiled-coil substantially reduced the number of the observed puncta, yet increased their size and intensity (*SI Appendix*, Fig. S5*C*). This suggests that the coiled-coil domains of MFP1 may promote the formation of protein hotspots. This is consistent with behavior of the soluble MFP1ΔTMD-YFP, which was present in a few large puncta (Fig. 2*C*).

### Membrane Association of MFP1 Is Not Required but Advantageous for Proper Granule Initiation.

**Fig. 3.**
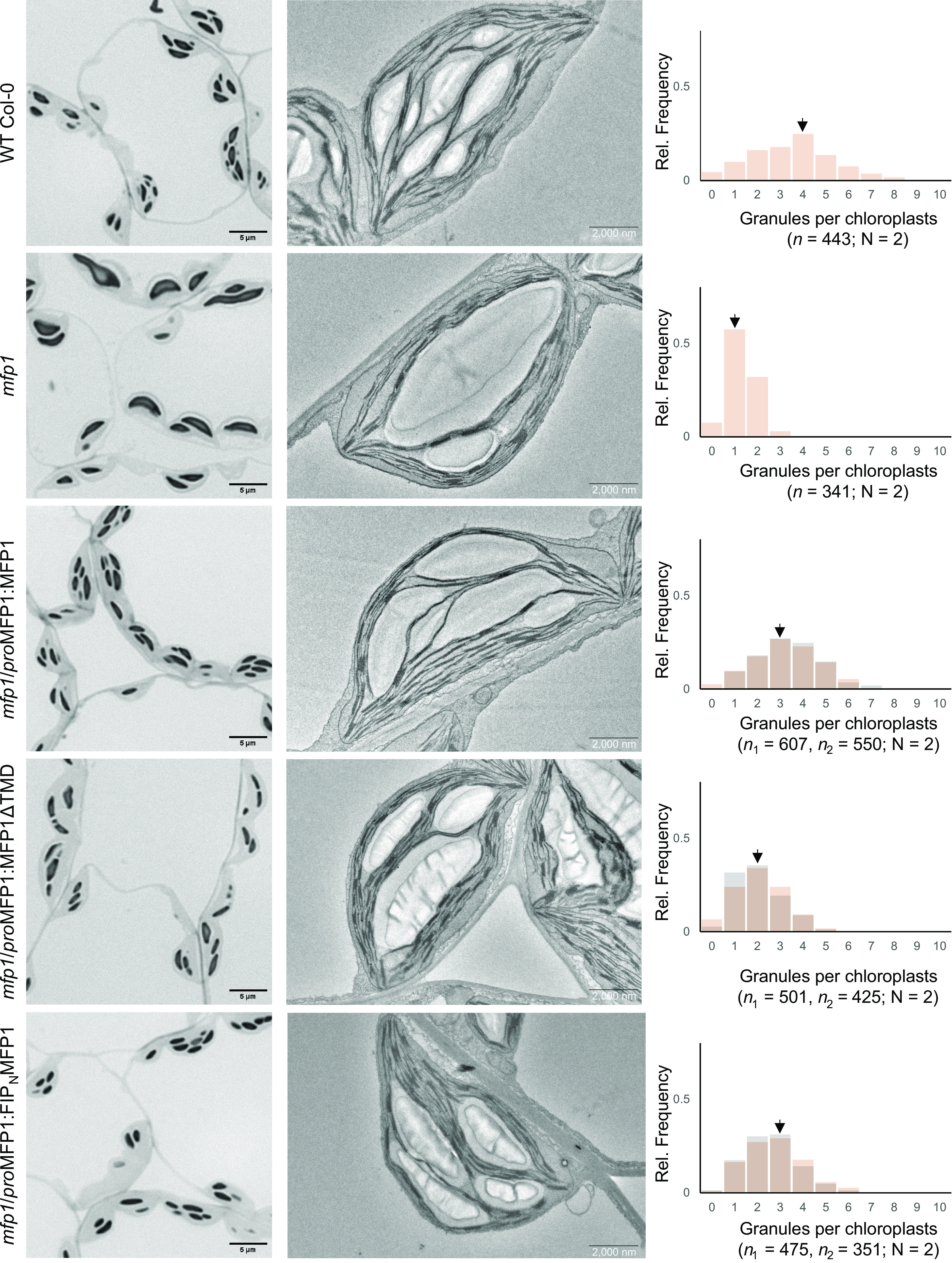
Thylakoid anchorage is important for MFP1-mediated starch granule initiation. (*Left* lane) Light microscopy (LM) images of toluidine blue stained semithin leaf sections, (*Middle* lane) TEM images of representative chloroplasts from leaf tissues harvested from the indicated transgenic lines at the EOD, and (*Right* lane) histograms of starch granule count per chloroplast section (n) counted for two biological replicates each (N). In the case of lines expressing different MFP1 variants, the resulting histograms of two independent transgenic lines for each construct are overlaid and displayed in orange and gray colors. Black arrows indicate the median. On the *Y* axis is the relative frequency of occurrence of the specified number of granules per chloroplast (*X* axis).

We noticed that the *mfp1* mutant phenotype was only partially complemented by expression of YFP-tagged MFP1, with the majority of chloroplast sections still containing only 1 to 3 granules (*SI Appendix*, Fig. S5*B*). This suggested either that the large fluorescent tag impairs MFP1 function or its expression under the ubiquitin promoter is inadequate. Therefore, we created additional transgenic lines expressing the same MFP1 versions, but devoid of tags and expressed under the control of the native *MFP1* promoter. Again, we expressed the proteins in the *mfp1* mutant background and selected two lines of each, showing similar MFP1 expression levels (as assessed by immunoblotting; *SI Appendix*, Fig. S6), for further analysis. We confirmed that the untagged version of MFP1 complemented the *mfp1* mutant phenotype well, with similar granule numbers per chloroplast as in WT plants (Fig. 3). However, this was not the case for lines expressing MFP1ΔTMD, which showed only partial complementation, with a median granule number of 2 per chloroplast section—higher than the *mfp1* knockout line, yet lower compared to MFP1 complemented lines. In contrast, FIP_N_MFP1 expression restored near-normal starch granule numbers (Fig. 3). Together, these data indicate that solubilized MFP1 can promote starch granule initiation, but for full efficacy, its membrane association is essential.

### Inner-Envelope-Localized MFP1 Results in Aberrant Starch Granule Formation.

**Fig. 4.**
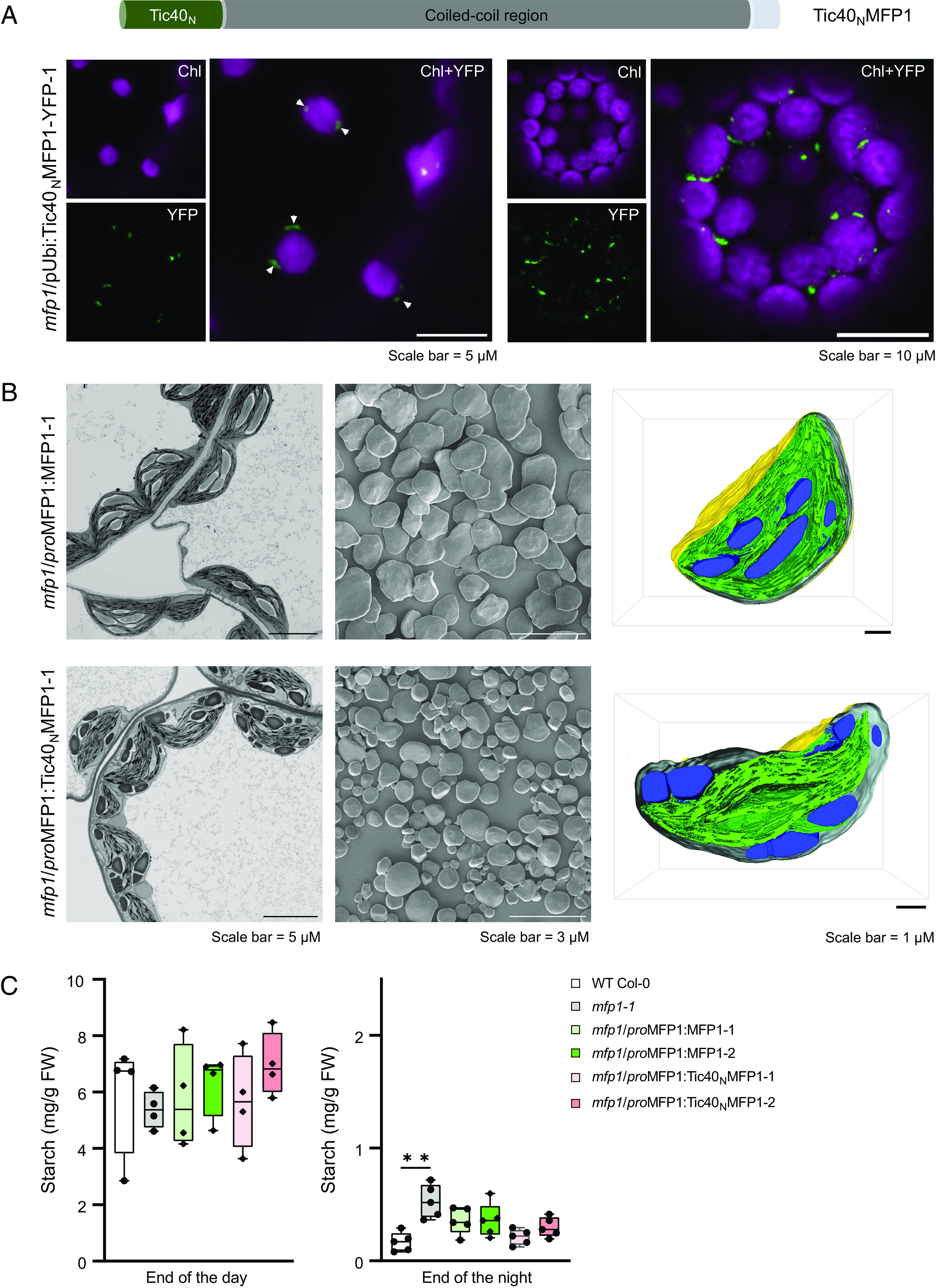
Directing MFP1 to the chloroplast envelope is sufficient to shift starch granule biogenesis near envelope. (*A*) Schematic representation of the Tic40_N_MFP1 variant (*Top*) and subchloroplast localization of Tic40_N_MFP, as assessed by confocal microscopy of leaf epidermal cells and mesophyll protoplasts isolated from leaf tissue of transgenic lines. Images are presented as chlorophyll channel (false color magenta), YFP channel (false color green), or the overlay of both channels. (*B*) A single 2-D SEM image (*Left*) segment obtained after FIB-milling of leaf section of the transgenic lines expressing either WT MFP1 or Tic40_N_MFP1 in the *mfp1* mutant background and SEM images (*Middle*) of purified starch granules from these lines. (*Right*) 3D reconstructions of manually segmented chloroplasts selected from multistack FIB-SEM images (Movies S1 and S2). In the 3D models, the chloroplast envelope, thylakoid membranes, and starch granules are colored in yellow-gray, green, and blue, respectively. (*C*) Starch quantification of transgenic lines expressing, MFP1 and Tic40_N_MFP1 with respective controls. Entire rosettes (4 to 5 biological replicates) were harvested at the EOD or EON, the starch therein extracted and measured using an enzyme-coupled assay after digestion to glucose. An unpaired *t* test was performed to determine any statistical significance. The significant difference with *P*-value < 0.05 is depicted with two asterisks.

We next sought to analyze whether association to chloroplast membranes other than thylakoids is sufficient for MFP1 to fulfill its function. To directly test this, we replaced the N-terminal signal peptide and transmembrane domain of MFP1 with the N-terminal bipartite signal sequence and transmembrane domain of a chloroplast inner envelope protein, TRANSLOCON AT THE INNER ENVELOPE MEMBRANE OF CHLOROPLASTS 40 (Tic40) (Fig. 4*A*). Envelope localization of Tic40 is achieved by a two-step process where it is first imported into the chloroplast via an envelope translocon and later integrated into the inner envelope, exposing its C-terminal region toward the stroma (16).

To test whether the Tic40 signal sequence directs MFP1 to the inner plastid envelope membrane, we first determined the localization of a YFP-tagged version of Tic40_N_MFP1, expressed under the control of the ubiquitin promoter in the *mfp1* mutant background. As expected, Tic40_N_MFP1-YFP localized to the periphery of the chloroplasts in epidermal cells and mesophyll protoplasts (Fig. 4*A*). Interestingly, the protein formed distinct puncta, consistent with its behavior when thylakoid-associated or soluble. To analyze starch-related phenotypes, we further created transgenic lines expressing untagged Tic40_N_MFP1 under the control of the native *MFP1* promoter in the *mfp1* mutant background. We observed most starch granules to have formed at the periphery of the chloroplasts in both independent transgenic lines (Fig. 4*B*). The number of these granules was higher compared to WT plants (*SI Appendix*, Fig. S7*A*), and they appeared smaller and more rounded, an impression that was confirmed by SEM and FC analyses of purified starch granules (Fig. 4*B* and *SI Appendix*, Fig. S7*B*). The total leaf starch content of plants expressing Tic40_N_MFP1, quantified at the EOD and EON, remained unchanged compared to WT and MFP1 complementation lines, showing that the diurnal pattern of starch metabolism was unaffected (Fig. 4*C*).

To acquire more detailed, in-situ information about the starch granules in the context of chloroplast membranes of the Tic40_N_MFP1 lines, we further deployed focused ion beam–SEM (FIB-SEM) and generated detailed three-dimensional reconstructions of the chloroplast ultrastructure and content (Fig. 4*B*). These data confirmed that while starch granules in the lines complemented with native MFP1 are formed in stromal spaces fully surrounded by thylakoid membranes (Movie S1), conversely in lines expressing Tic40_N_MFP1, granules were formed next to the envelope membranes (Movie S2).

### Envelope-Localized MFP1 Changes the Subchloroplast Location of PTST2 and SS4.

**Fig. 5.**
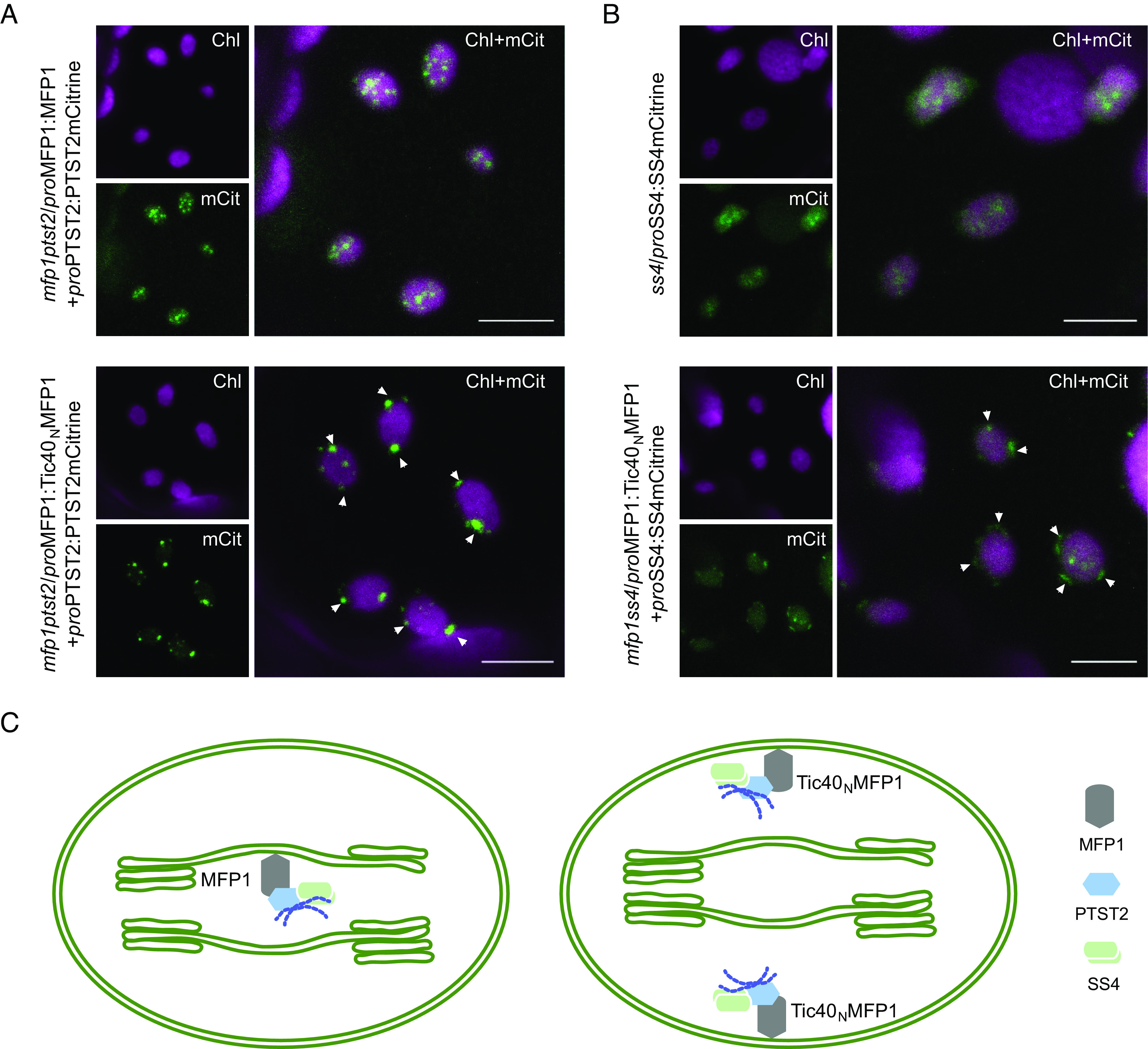
Tic40_N_MFP1 is sufficient to redirect PTST2 and SS4 to the chloroplast envelope. (*A*) PTST2-mCitrine localization in transgenic lines expressing either WT MFP1 or Tic40_N_MFP1, as assessed by confocal microscopy of epidermal cell chloroplasts. (*B*) SS4-mCitrine localization in transgenic lines expressing either WT MFP1 or Tic40_N_MFP, as assessed by confocal microscopy of epidermal cell chloroplasts. (Scale bar, 5 µm.) (*C*) A cartoon indicating the expected relocalization of PTST2 and SS4 in chloroplast based on its interaction with MFP1. The glucan chains (shown in blue) bind to PTST2 and serve as substrates for granule initiation at a specific location within chloroplasts. (*Left*) Thylakoid-localized MFP1 and (*Right*) envelope-localized MFP1.

It was previously shown that PTST2 associates with thylakoid membranes and that this localization depends on MFP1, as in the absence of MFP1, PTST2 remains exclusively localized within the chloroplast stromal fraction (7). Furthermore, PTST2-mCitrine localized to multiple distinct puncta within the chloroplasts, colocalizing with MFP1, whereas its localization is restricted to only a few puncta in *mfp1* knockout lines (7). To determine whether the envelope-localized MFP1 variant was sufficient to relocalize PTST2 within chloroplasts, we transformed the *mfp1 ptst2* double mutant with constructs encoding either MFP1 and PTST2-mCitrine or Tic40_N_MFP1 and PTST2-mCitrine (expressed from the native *MFP1* and *PTST2* promoters, respectively). These lines displayed phenotypes similar to those observed for the corresponding *mfp1* complementation lines expressing MFP1 or Tic40_N_MFP1, reconfirming that tagged PTST2 is functional (7) (Fig. 5*A* and *SI Appendix*, Fig. S8*A*). We then analyzed the subchloroplast location of PTST2-mCitrine via confocal microscopy in leaves of the resultant transformants. Since the expression levels of PTST2-mCitrine driven from the native promoter were relatively low, we focused mainly on epidermal cell chloroplasts. As expected, when expressed with MFP1, PTST2-mCitrine formed distinct punctate structures within the chloroplasts. When expressed with Tic40_N_MFP1, these punctate structures shifted to the periphery of chloroplasts (Fig. 5*A*).

We further investigated whether the subchloroplast localization of SS4, a crucial protein involved in starch granule initiation, also changes in plants expressing Tic40_N_MFP1. For this, we introduced constructs encoding either SS4-mCitrine alone or Tic40_N_MFP1 along with SS4-mCitrine, driven by their native promoters, into *ss4* or *mfp1ss4* lines, respectively. These lines displayed phenotypes similar to those observed for the corresponding *mfp1* complementation lines, confirming that tagged SS4 is functional (*SI Appendix*, Fig. S8*B*). The subcellular distribution of SS4-mCitrine in these lines was examined by confocal microscopy. In the lines expressing SS4-mCitrine alone, the protein localized primarily in the stroma within some discrete puncta, whereas in the lines expressing the envelope-localized MFP1, the SS4-mCitrine puncta localized at the chloroplast periphery (Fig. 5*B* and *SI Appendix*, Fig. S8*C*). This demonstrates that MFP1 is a key driver of PTST2 and SS4 localization within chloroplasts.

## Discussion

Chloroplasts are specialized structures within plant cells that contain around 3,000 different proteins and a complex network of thylakoid membranes (17). Within this highly organized environment, the process of forming starch granules presents unique challenges. One of the challenges involves the need to achieve spatiotemporal separation between the enzymatic machinery responsible for starch synthesis and the machineries executing other essential functions of the chloroplast. Second, the semicrystalline starch granules themselves may potentially interfere with diffusional processes or photosynthetic functions of the thylakoid membranes. Third, starch granules could potentially interfere with the repeated divisions of the chloroplasts that occur as photosynthetic tissues develop. Therefore, it is perhaps unsurprising that there are mechanisms in place to determine the number of starch granules, and where exactly they form (11). The recent discovery of a myriad of novel protein players has underlined the importance and complexity of this phenomenon (5–8), even if many aspects of their respective molecular actions remain elusive.

There is good evidence that selective affinity for certain malto-oligosaccharide species is important for the initiation of granule biogenesis. This has been experimentally demonstrated for PTST2, which possesses a carbohydrate-binding module (CBM) and probably provides short glucan chains, suitable for elaboration into a granule initial, to its interaction partner, SS4. Similarly, the PTST2 homologue, PTST3, also features a CBM and may serve a related function (6). In contrast, the roles of the two coiled-coil domain-containing proteins MFP1 and MRC remain largely elusive. Apart from engaging in protein–protein interactions and apparently promoting granule initiation (7, 8), their molecular function has not been understood in detail.

Our data on MFP1, the only known starch granule initiation protein that harbors a thylakoid membrane anchor, suggests a dual role, first in promoting starch granule initiation and second in determining the location of granule initiation within chloroplasts. The number of granules per chloroplast increased when MFP1 was overexpressed, while the overall morphology and positioning of starch granules on a subchloroplast level remained largely unchanged (Fig. 1). In comparison to the overexpression of PTST2, which not only increased granule counts dramatically but also let them form in unexpected areas, the effect of MFP1 overexpression was subtler. This may be explained by inherent functional differences between these two proteins. The ability of PTST2 to interact with short glucan chains and the granule initiation enzyme, SS4 directly contributes to the formation of granule initials. The role of MFP1, on the other hand, appears to be more of an indirect one—to guide and localize PTST2 and consequently the other initiation proteins to specific places. Based on publicly available proteomics data, MFP1 appears to be the most abundant protein among those involved in the starch granule initiation process, while the abundance of PTST2 is comparatively low (*SI Appendix*, Fig. S9). Thus, when MFP1 is overexpressed, PTST2 might be a limiting factor restricting the initiation of starch granules at sites between the thylakoid membranes. In contrast, overexpression of PTST2 may overwhelm the capacity of endogenous thylakoid-localized MFP1 to direct it to suitable locations, thereby leading to uncontrolled granule initiation throughout the stroma (Fig. 1). Supporting this notion, the simultaneous overexpression of MFP1 and PTST2 was shown to further increase the total number of starch granules per chloroplast while ensuring the correct localization of granule formation (*SI Appendix*, Fig. S2). Nevertheless, the significance of other initiation components, for example, MRC, SS5 and PTST3 in determining granule numbers cannot be overlooked. In these initiation mutants, even though the endogenous PTST2 and MFP1 are still expressed (7), the granule count per chloroplast go down.

The observed punctate localization of MFP1, whether associated with the thylakoids, the inner envelope, or free in the stroma, is a feature also evident in other proteins involved in starch granule initiation for example MRC and SS5 (5, 7). This pattern suggests that the process of starch granule initiation involves a regulated subcompartmentalization of the plastid’s stroma, bringing together multiple factors simultaneously. Previous studies have shown that membrane-less compartmentalization can be mediated via biomolecular condensation, resulting in liquid–liquid phase separation (LLPS) (18, 19). It will be exciting to investigate whether proteins involved in granule initiation engage in this type of interaction, and if so, which combination of protein factors is required. The presence of intrinsically disordered regions—a hallmark of proteins driving LLPS—in both MFP1 and PTST2 proteins indicates the possibility of this phenomenon, occurring specifically at the stroma/thylakoid interface.

When MFP1 is dissociated from the thylakoids, the MFP1ΔTMD protein localizes to a reduced number of puncta compared to MFP1 and only partial complementation of the *mfp1* phenotype was observed, although its total abundance was not altered (Fig. 3). However, when MFP1 was reassociated with thylakoid membranes using a distinct thylakoid targeting mechanism, the number of puncta and accordingly the number of starch granules per chloroplast increased (Figs. 2 and 3). This suggests that equitable distribution of MFP1 within chloroplasts and the allocation of resources required for starch granule formation is dependent on MFP1 being anchored to thylakoid membranes.

Lastly, we found that the relocalization of MFP1 to the plastid’s inner envelope membrane, via its attachment of the Tic40 N terminus, was adequate to induce the formation of most starch granules in direct proximity to the envelope (Fig. 4). This is remarkable, since it shows that the location of this one protein is sufficient to redirect the action of the majority if not the entire starch biosynthetic apparatus, which consists of over 10 other proteins (2). It is interesting to note that when starch granules were produced in this aberrant location, their sizes and morphologies were changed relative to WT granules (Fig. 4*B* and *SI Appendix*, Fig. S7). The underlying reason for this is unclear, but it could be attributed to aberrant granule initial coalescence events near the envelope membrane or impaired anisotropic granule expansion due to the absence of guiding forces from opposing thylakoid membranes (1). Indeed, it was recently reported that aberrant thylakoid organization can lead to changes in the number, shape, and size of starch granules (20). While these speculations hold merit, we should also consider the possibility that not all of the necessary factors of the starch granule initiation and/or biosynthesis machinery are equally well redirected to the envelope with envelope-localized MFP1. While we have shown that two key starch granule initiation proteins, PTST2 and SS4 are relocalized in Tic40_N_MFP1 lines (Fig. 5), but further analyses will be required to determine the influence on the other granule initiation proteins.

## Materials and Methods

### Plant Material and Growth Conditions.

Plants were grown under 12-h light/12-h dark diel cycles, with 150 µmol m^−2^ s^−1^ light, at 20 °C temperature and 65% relative humidity in a Percival Plant Growth Chamber (CLF Plant Climatics) equipped with red LED panels and fluorescent bulbs. The previously characterized Arabidopsis T-DNA insertion mutant lines *mfp1*-1 and *ptst2*-7 were used (described in Seung et al., 7); both are in the Columbia-0 wild-ecotype background. The *mfp1ptst2* and *mfp1ss4* double mutant lines were generated by crossing the respective single mutants and isolating homozygous double mutants by PCR-based genotyping of the resultant F2 population. The single mutants were obtained from Nottingham Arabidopsis Stock Center with accession numbers SALK_124298 (*mfp1-1*), SALK_73591 (*ptst2-7*), GABI_290D11 (*ss4*) and has been characterized earlier (7).

### Cloning and Plant Transformation.

Gene coding sequences for *MFP1* (AT3G16000) and *PTST2* (AT1G27070), amplified as described previously (6, 7), were cloned into the Gateway entry vector pDONR221, thereby generating *pDONR221:MFP1* and *pDONR221:PTST2*. Coding sequences for the N-terminal 82 and 125 amino acids of FIP (AT5G02160) and Tic40 (AT5G16620), respectively, were amplified from Arabidopsis cDNA preparation. Restriction-free (RF) cloning (21) was used to generate *pDONR221:MFP1ΔTMD*, *pDONR221:FIP_N_MFP1,* and *pDONR221:Tic40_N_MFP1* by either deleting the coding sequences for amino acids 71 to 146 of MFP1 encoding the predicted thylakoid signal peptide and transmembrane domain (for MFP1ΔTMD) or replacing complete N-terminal amino acids 1 to 146 (for *FIP_N_MFP1 and Tic40_N_MFP1*). The sequences are available in *SI Dataset*. For determining the subcellular localizations of the MFP1 variants, the pDONR221 entry vectors carrying the gene fragments described above were recombined into the pUBC-YFP destination vector (22) using LR clonase II (Thermo Fisher Scientific) in a Gateway cloning reaction.

The native promoter of *MFP1* (1,704 bp upstream of the translation start codon) was amplified using Arabidopsis genomic DNA as template and cloned in a gateway entry vector, generating *pDONRP4P1r:proMFP1*. For generation of tag-free complementation lines, the entry vectors carrying *MFP1* variants as described above were modified via RF cloning so as to insert a stop codon. They were then subcloned into the binary plant expression vector R4pGWB607 (23) together with *pDONRP4P1r:proMFP1*. For the generation of MFP1 overexpression lines, the *pDONR221:MFP1* vector with a stop codon was recombined with the plant expression vector pGWB608 (23) via Gateway LR cloning. The PTST2 overexpression lines used in this work were generated previously (6).

Dual site Gateway cloning (24) was utilized to clone two gene construction into a single plant expression vector. For generation of double overexpression lines, the entry vector carrying a Ubiquitin 10 (pUBI) promoter of Arabidopsis was recombined into modified R4pDD607-mCitrine vector together with *pDONR221:PTST2*, generating *R4pDD607-pUbi:PTST2mCitrine*. This vector was then recombined into the plant expression vector R4pGWB6607-MD8 together with *pDONRP4P1r:CaMV35S* and *pDONR221:MFP1, carrying a stop codon.* Similarly for generation of transgenic lines to determine subchloroplast localization, the entry vector carrying the native promoter of *PTST2* (7) was recombined into the modified R4pDD607-mCitrine vector together with *pDONR221:PTST2*, generating *R4pDD607-proPTST2:PTST2mCitrine*. This vector was then recombined into the plant expression vector R4pGWB6607-MD8 together with *pDONRP4P1r:proMFP1* and *pDONR221:MFP1* or *pDONR221:Tic40_N_MFP1*, each carrying a stop codon.

For expression of fluorescently tagged SS4 protein in plants, the genomic DNA sequence of Arabidopsis SS4 (AT4G18240) carrying its promoter, 5′ UTR, exons and introns was cloned into the entry vector pDONR221, recombined into modified R4pDD607-mCitrine vector together with a dummy vector to replace R4-R1 gateway sites, generating *R4pDD607-SS4mCitrine.* This vector was then recombined into the plant expression vector R4pGWB6607-MD8 together with *pDONRP4P1r:proMFP1* and *pDONR221:Tic40_N_MFP1*, carrying a stop codon. For expression of SS4-mCitrine only, the entry vector carrying SS4 genomic fragment was recombined into binary expression vector pH7m35GW,0 together with pDONR-mCitrine and a dummy vector to replace R4-R1 gateway sites.

Binary plant expression vectors were transferred into the *Agrobacterium tumefaciens* strain GV3101 (pMP90) (25). Transient transformation of *Nicotiana benthamiana* leaf tissue was performed as described previously (26). Arabidopsis plants were transformed using a floral dip method as described by Davis et al. 27.

### Leaf Protein Extraction and Separation.

For extraction of total proteins from leaves, 9-mm leaf discs were homogenized in 100 µL of Laemmli buffer (50 mM Tris-HCl, pH 6.8, 2% [w/v] sodium dodecyl sulfate (SDS), 100 mM dithiothreitol (DTT), 3% [v/v] glycerol, and 0.005% [w/v] bromophenol blue), heated at 95 °C for 5 min and clarified by centrifugation (19,000 g, 5 min, 20 °C). The supernatants were collected and used for analysis via SDS-Polyacrylamide gel electrophoresis. Extraction of total, soluble, and membrane-bound proteins was performed as described previously (7).

### Recombinant Protein Expression and Immunoblotting.

The coding sequence of Arabidopsis *MFP1*, excluding the N-terminal 146 amino acids encoding the predicted transit peptide and transmembrane domain, was cloned into the bacterial expression vector pPROEX HTb (Invitrogen) using *Bam*HI and *Sal*I restriction endonucleases. The construct was then transformed into *Escherichia coli* Arctic Express (Agilent) cells. Protein expression and purification was performed as described in David et al. (28). The HisTrap HP 1 mL columns (Cytiva Life Sciences) were used to purify the histidine-tagged MFP1 and the proteins were eluted using elution buffer (50 mM Tris–HCl pH 7.5, 300 mM NaCl, 40 mM imidazole, 2 mM DTT) with a linear gradient of imidazole concentration increasing up to 500 mM. The eluted protein fractions containing MFP1 were concentrated using Amicon Ultra centrifugal units with a molecular size cutoff of 10 kDa and buffer exchanged into phosphate-buffered saline (137 mM NaCl, 2.7 mM KCl, 10 mM Na_2_HPO_4_, and 1.8 mM KH_2_PO_4_) using a NAP25 column.

For antibody production, rabbits were immunized by Eurogentec (Belgium). Antibodies specifically recognizing MFP1 were purified by affinity purification against MFP1 antigen. Immunoblotting with the purified, diluted primary α-MFP1 antibodies (1:2,000) was performed as described previously (5).

### Confocal Microscopy.

For subcellular protein localization, a confocal laser scanning system Zeiss LSM780, with inverted objectives, was utilized. Either 514-nm (YFP and mCitrine) argon or 633-nm (chlorophyll) helium-neon lasers were used as the light source. Sequential image acquisition was done using filters ranging from 526 to 624 nm (for YFP and mCitrine) and 647 to 721 nm (for chlorophyll). At least 10 regions of interest were imaged per leaf/plant sample. To look at epidermal cells, a section from the middle region of a mature leaf was excised and mounted on a slide with the adaxial side facing the 40× water objective. Protoplasts for the imaging of mesophyll cells were generated using the Tape-Arabidopsis Sandwich method (29) and placed on a 35 mm µ-Dish (ibiTreated, 1.5 polymer coverslip) for imaging.

### Light and Electron Microscopy.

For counting starch granule numbers per chloroplast, leaf sections were chemically fixed at the EOD using 2.5% [v/v] glutaraldehyde and 2% [v/v] formaldehyde, stained with osmium tetroxide, and embedded into Spurr (Polysciences, Inc.) epoxy resin after a series of ethanol mediated dehydration steps as described earlier (5). Semithin sections (0.5 µm) were cut using a microtome, mounted on coverslips, and stained with toluidine blue. Image acquisition was done with a Zeiss Axio Imager.Z2 imaging system equipped with 100× oil immersion objective lens.

The same resin blocks were used for electron microscopy. In this case, ultrathin (70 nm) sections were cut using a diamond knife, mounted on a copper grid, stained with 2% [w/v] uranyl acetate and Reynold’s lead citrate, and imaged with a Morgagni 268 microscope (FEI) as described previously (30).

### FIB-SEM.

Leaf sections were chemically fixed at the EOD and stained and embedded into Durcupan™ ACM epoxy resin (Sigma-Aldrich) as described in Bürgy et al. ( 1). A Thermo Fisher Scientific Helios 5 UX DualBeam system was used for FIB-milling (30 kV, 0.75 nA), and images were acquired (2 kV, 40 nA) with an Elstar in-column SE/BSE detector in immersion mode. Automated stack acquisition was performed using the Auto Slice & View 4 (Thermo Scientific) software. For segmentation of chloroplast features, Amira software (Thermo Scientific) was utilized.

### Starch Extraction and Quantification.

Whole rosettes from 35-d-old plants were harvested at the EOD or EON into tubes containing 10 heavy metal beads (5 mm), snap-frozen in liquid nitrogen, and stored at −80 °C until use. Plant material was ground to powder in a Geno Grinder 2010* (HORIBA) by shaking the tubes 4 × 1 min at 1,500 rpm with cooling in liquid nitrogen in between. The grounded tissue powder was suspended in 0.7 M perchloric acid, the homogenate was clarified by centrifugation (10 min, 3,000 g, 4 °C), and the pellet containing the starch was washed once in water and four times in 80% [v/v] ethanol. The pellet was then dried, resuspended in water, and stored at −20 °C until further processing. Starch was quantified using an enzymatic assay described previously (31).

### Starch Granule Purification and SEM.

Leaf tissues were harvested at the EOD and placed into 1.5-mL Eppendorf tubes together with 3 glass beads (4 mm), immediately frozen in liquid N_2_, and milled twice for 30 s each using a Retsch mixer mill at 30 oscillations s^−1^. The milled powder was then mixed via vortexing with 900 μL starch extraction medium (50 mM Tris-HCl, pH 8, 0.2 M ethylenediaminetetraacetate, 0.5% [v/v] Triton X-100, and 0.8% [w/v] SDS) and insoluble material collected by centrifugation (4,500 g, 5 min, 20 °C) to collect the pellet. The pellet was washed twice with 900 μL water and resuspended in 500 μL water. A 96-well filter plate with polyethylene terephthalate frit (20 μm pore size; Fisher Scientific) was used to purify the small granules away from larger cell wall debris. Wells were washed with 100 μL of water to ensure all starch was collected.

For SEM, chips sliced from Si-wafers were cleaned in ethanol, negatively glow discharged, and coated with Poly-L-Lysine. Samples were applied and chips were mounted to SEM-stubs with silver glue, air dried, sputter coated with 5 nm platinum (Pt)/palladium (Pd), and imaged with a Thermo Fischer Scientific Magellan 400 at 2 kV, 50 pA. Images were acquired 0° top down or at 50° tilt with an ETD detector, WD 4 mm.

### FC.

Purified starch samples were diluted 1:20 in water and analyzed using a CytoFLEX (Beckmann Coulter). First, samples were mixed by orbital shaking (1,500 rpm for 6 s, acceleration time: 1 s), and measurements were performed using default excitation power settings. The flow rate was set between 3,000 and 6,000 particles per second with a stop at 100,000 counts. The correlation of particle diameter and forward scattering (FSC) values was adjusted as described in Thieme et al. (12)

## Supplementary Material

Appendix 01 (PDF)

Dataset S01 (XLSX)

Movie S1. 3D-reconstruction of a single chloroplast, manually segmented from electron micrograph z-stack obtained after FIB-SEM of leaf tissue expressing wild type MFP1 in *mfp1* line. The chloroplast envelope, thylakoid membranes, and starch granules are colored in yellow-gray, green, and blue, respectively, in the 3D-models. (supplement to Fig. 4)

Movie S2. 3D-reconstruction of a single chloroplast, manually segmented from electron micrograph z-stack obtained after FIB-SEM of leaf tissue expressing Tic40^N^MFP1 in *mfp1* line. The chloroplast envelope, thylakoid membranes, and starch granules are colored in yellow-gray, green, and blue, respectively, in the 3D-models. (supplement to Fig. 4)

## Data Availability

All study data are included in the article and/or supporting information.
