# Supplementary material for: MFP1 defines the subchloroplast location of starch granule initiation
Source: Proc Natl Acad Sci U S A. 2024 Jan 8;121(3):e2309666121. doi: 10.1073/pnas.2309666121 (PMC10801857; doi:10.1073/pnas.2309666121)
Supplement: Supplementary file 1 — Appendix 01 (PDF) [file pnas.2309666121.sapp.pdf]

PNAS

Supporting Information for

MFP1 defines the sub-chloroplast location of starch granule initiation

Mayank Sharma[1]*, Melanie R. Abt[1], Simona Eicke[1], Theresa Ilse[1], Chun Liu[1], Miriam S. Lucas[2], Barbara Pfister[1], Samuel C. Zeeman[1]*

*Address for correspondence- szeeman@ethz.ch; masharma@ethz.ch

This PDF file includes:

Figures S1 to S9
Legends for Movies S1 to S2

Other supporting materials for this manuscript include the following:

Movies S1 to S2

Figure S1

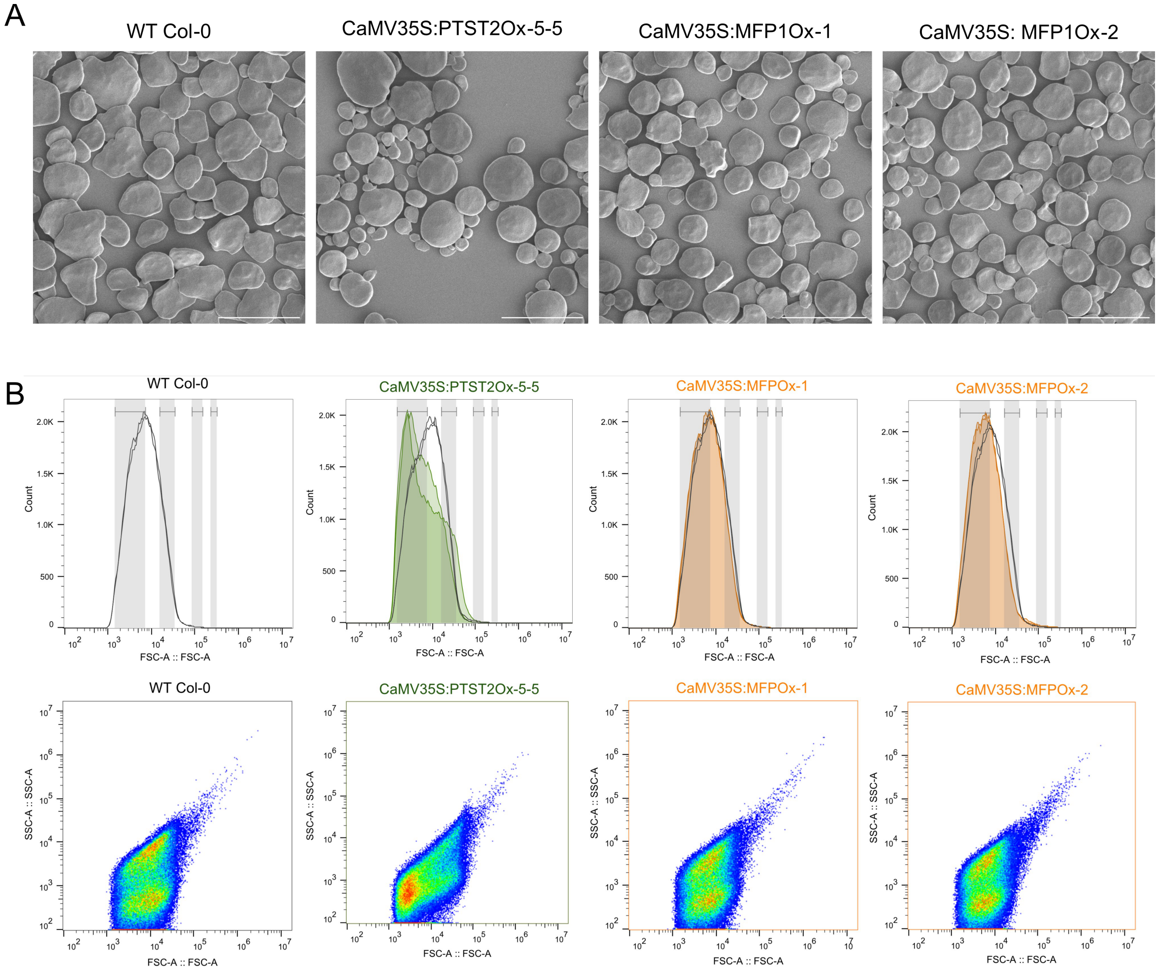

Fig. S1. Overexpression of PTST2 or MFP1 affects starch granule size and morphology.

(A) SEM images of purified starch granules from leaf tissue of the indicated lines harvested at the EOD. (supplement to Fig. 1) Scale bar=5 μM.

(B) Flow cytometry (FC) analysis of purified starch granules in two biological replicates. *(upper panels)*: histograms of the forward scatter (FSC-A) in overexpression lines (green and orange) in relation to the wild-type distribution (underlaid by a black line). The first grey bar in the histogram corresponds to granule sizes ranging from 0.1-0.5μm, the second one is from 1-2 μm, the third one from 4-6 μm and the last one from 8-10μm. A slight shift towards smaller size is observable in the FSC of granules corresponding to MFPOx-2 line whereas a significantly more pronounced shift was evident with PTST2 overexpression. *(bottom panels)*: Two-dimensional scatter plot of forward scatter (FSC-A) against sideward scatter (SSC-A). Two distinct density centers in the plots indicate the presence of typical lenticular shaped starch granules while a single density center indicates the presence of rounder than lenticular shaped granules. (supplement to Fig. 1).

Figure S2

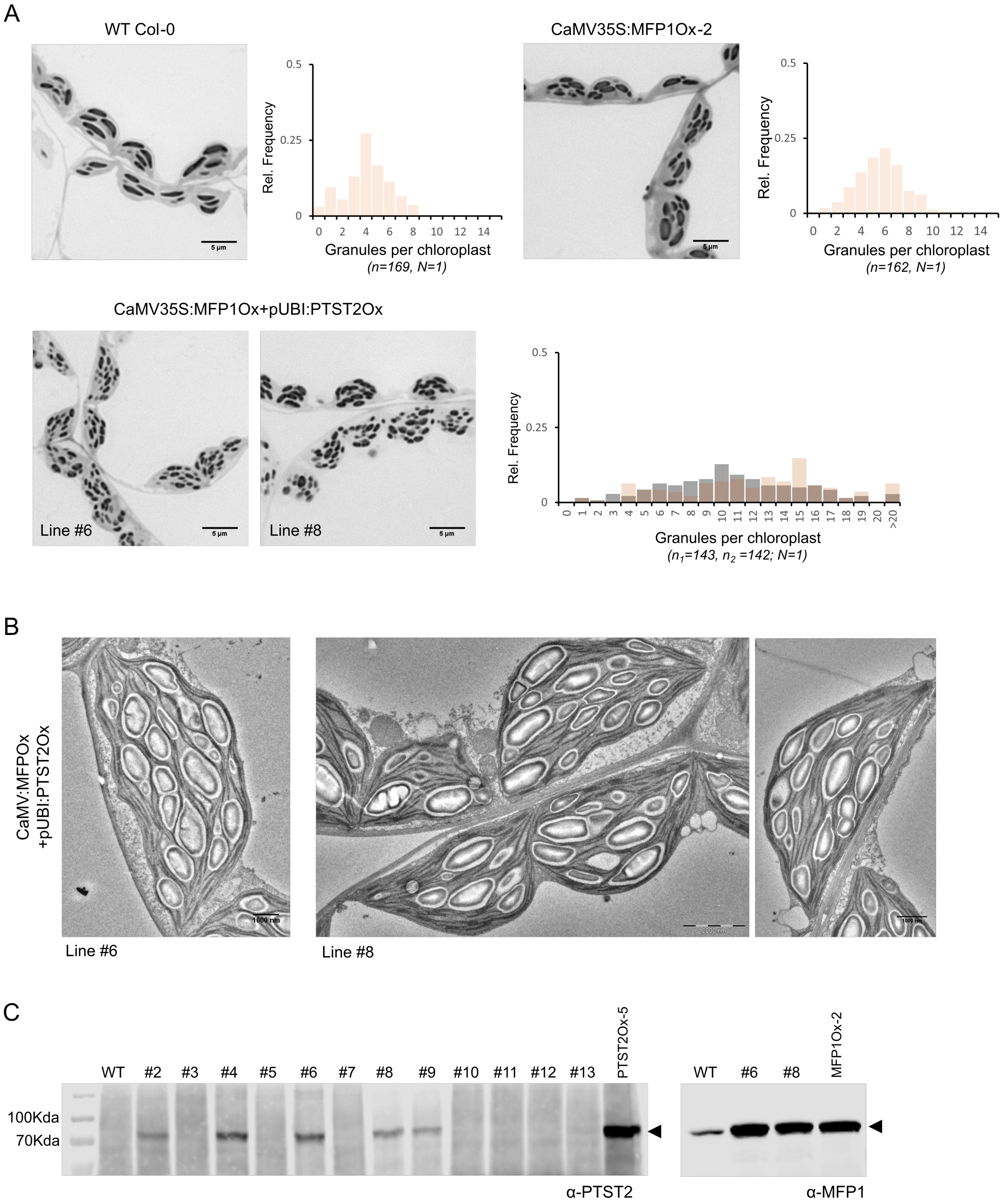

Fig. S2. Simultaneous overexpression of MFP1 and PTST2 in wild-type plants leads to dramatic increase in granule numbers.

(A) LM images of semi-thin sections obtained from fixed and embedded leaf tissues harvested at the EOD. Starch granules are visible as dark structures inside the chloroplasts. Manual count of starch granules per chloroplast section (n) are displayed as histograms. Histograms represent starch granule counts from indicated lines in orange color, the overlaid histograms of two independent double overexpresser lines are depicted in orange and gray colors.

(B) TEM images of chloroplasts in leaves of the indicated lines harvested at the EOD.

(C) Overexpression of PTST2-mCitrine and MFP1 was confirmed by immunoblotting using antibodies raised against PTST2 or MFP1 protein. Gels were loaded on an equal fresh weight basis. Protein extracts from lines overexpressing single constructs (PTST2Ox5-5 and MFP1Ox-2) were used for comparison.

Figure S3

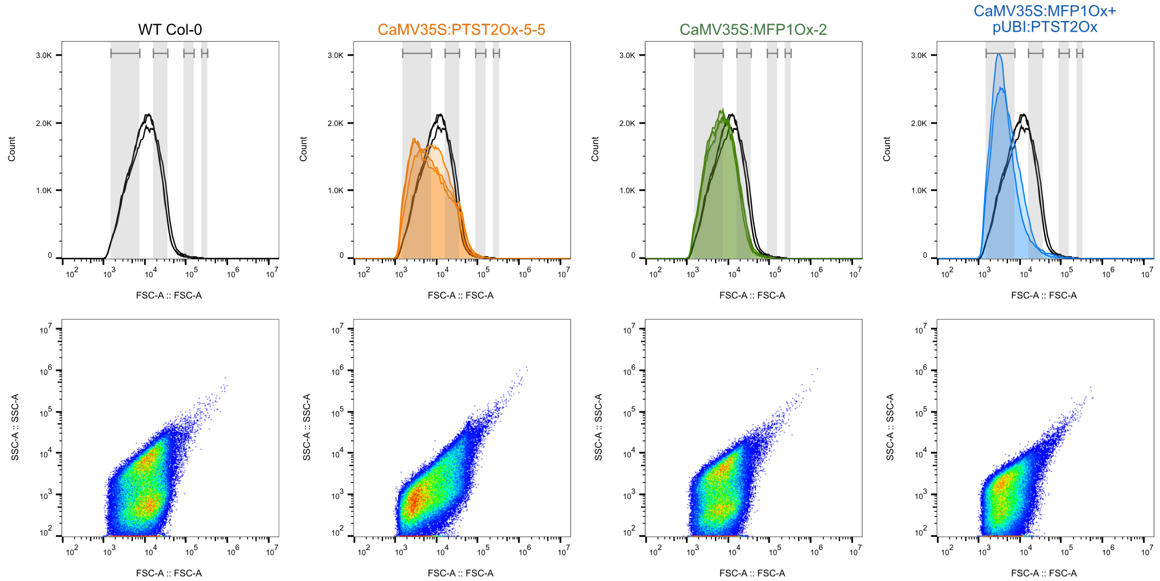

Fig. S3. Flow cytometry (FC) analysis of purified starch granules from lines overexpressing MFP1 and PTST2.

(upper panels): histograms of the forward scatter (FSC-A) in overexpression lines (orange and green) in relation to the wild-type distribution (underlaid by a black line). The first grey bar in the histogram corresponds to granule sizes ranging from 0.1-0.5µm, the second one is from 1-2 µm, the third one from 4-6 µm and the last one from 8-10µm. A significantly more pronounced shift towards smaller size was evident with overexpression of both MFP1 and PTST2. *(bottom panels)*: Two-dimensional scatter plot of forward scatter (FSC-A) against sideward scatter (SSC-A). Two district density centers in the plots indicate the presence of typical lenticular shaped starch granules while a single density center indicates the presence of rounder than lenticular shaped granules. There was no or little effect on shape of starch granules in double overexpresser lines.

Figure S4

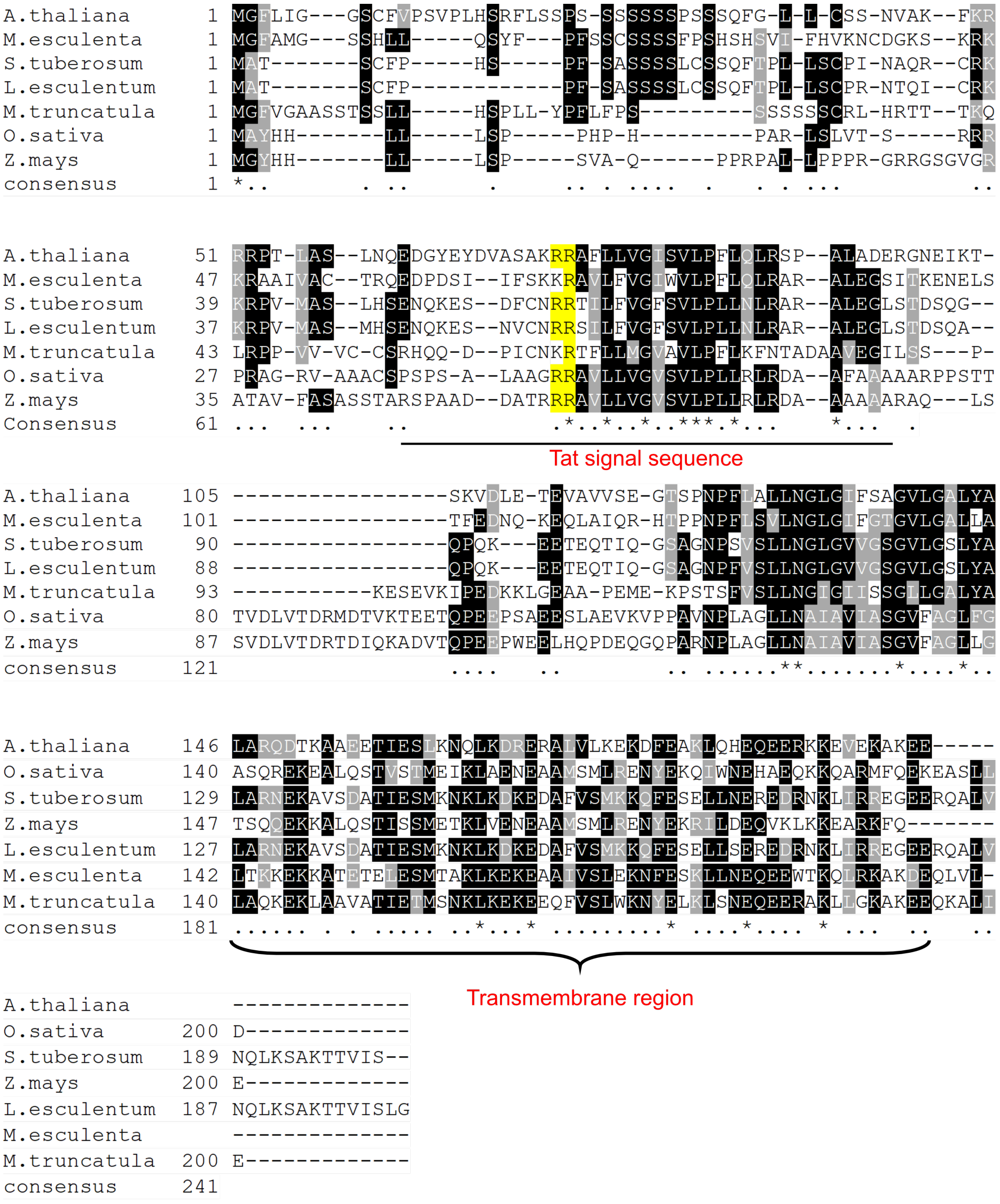

Fig. S4. Alignment of N-terminal sequences from MFP1 orthologues.
Shown is a ClustalOmega alignment of the first 200 amino acid residues of MFP1 sequences from seven different plant species. T-Coffee multiple sequence alignment program was used to create alignment. A yellow background indicates the twin arginines, and a black line highlight the Tat signal sequence. The transmembrane region is indicated with a bracket.

Figure S5

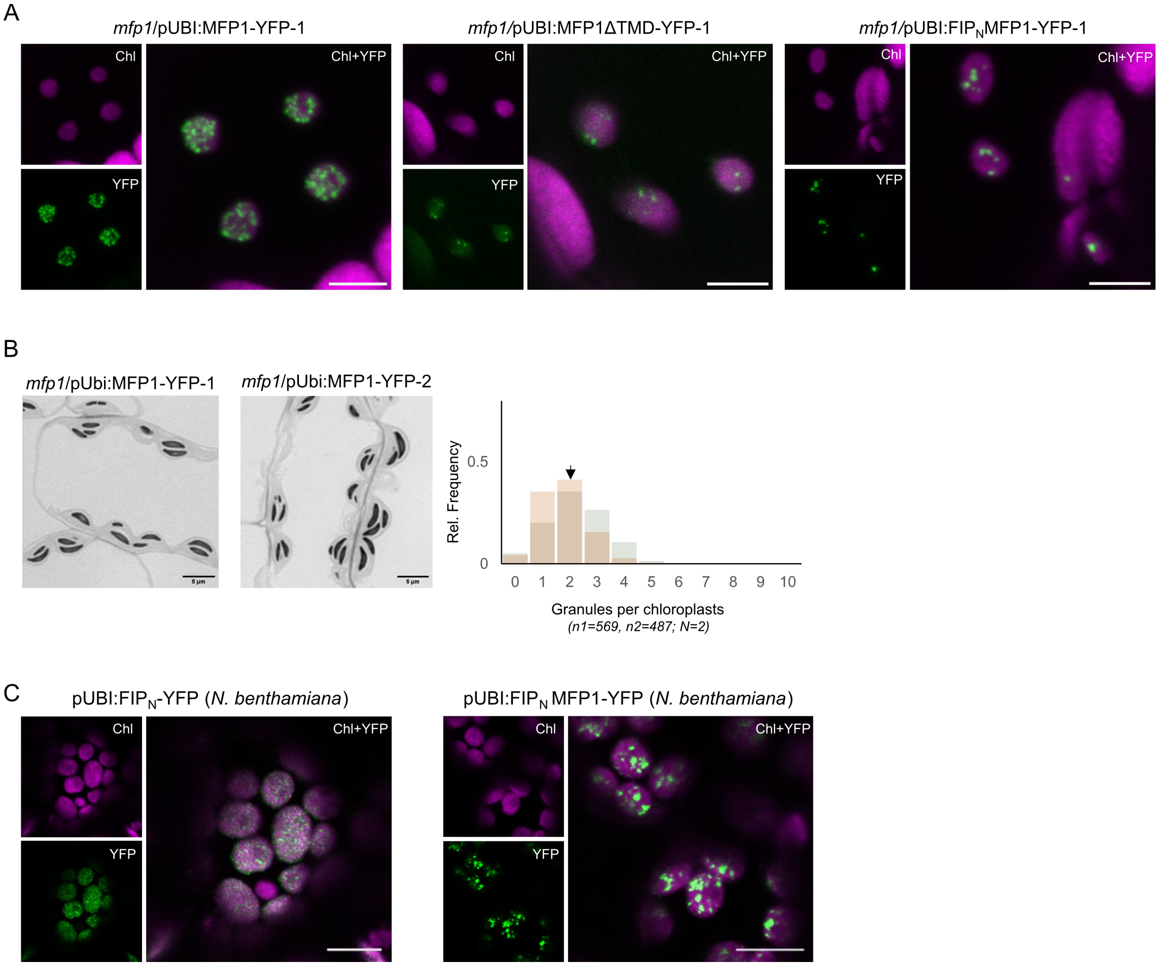

Fig. S5. Sub-chloroplast localization pattern of the expressed MFP1 variants.
(A) Confocal microscopy of leaf epidermal cells of Arabidopsis stable transgenic lines expressing MFP1-YFP, MFP1ΔTMD-YFP and FIP$_N$MFP1-YFP, under the control of ubiquitin promoter. (supplement to Fig. 2). Scale Bar=5 µM.
(B) Complementation of the *mfp1* mutant phenotype by expression of MFP1-YFP under the ubiquitin promoter is incomplete. LM image (left) and histograms of starch granules counts per chloroplast section (n) in two biological replicates (N) (right). The YFP-tagged version of MFP1 does not fully restore the *mfp1* mutant phenotype. Black arrow indicates median of histogram. Refer to Fig. 3 for the granule counts of wild-type and *mfp1* knockout lines. Harvesting and further processing of the corresponding samples were performed in a single batch.
(C) The distribution pattern of FIP$_N$MFP1-YFP is influenced by both the thylakoid localization and the MFP1 coiled-coil region. Confocal microscopy of mesophyll cell chloroplast of *Nicotiana benthamiana* transiently expressing chimeric proteins. (left) FIP$_N$-YFP (N terminal 1-82 aa of FIP fused to YFP) alone adopts a punctate distribution pattern inside the chloroplasts, (right) when fused to MFP1 in FIP$_N$MFP1-YFP, the number of puncta decreases, but their fluorescence intensity is increased. Images are presented as either chlorophyll channel (false color magenta), YFP channel (false color green) or the overlay images of both channels (merge). Scale bar=10 µM.

Figure S6

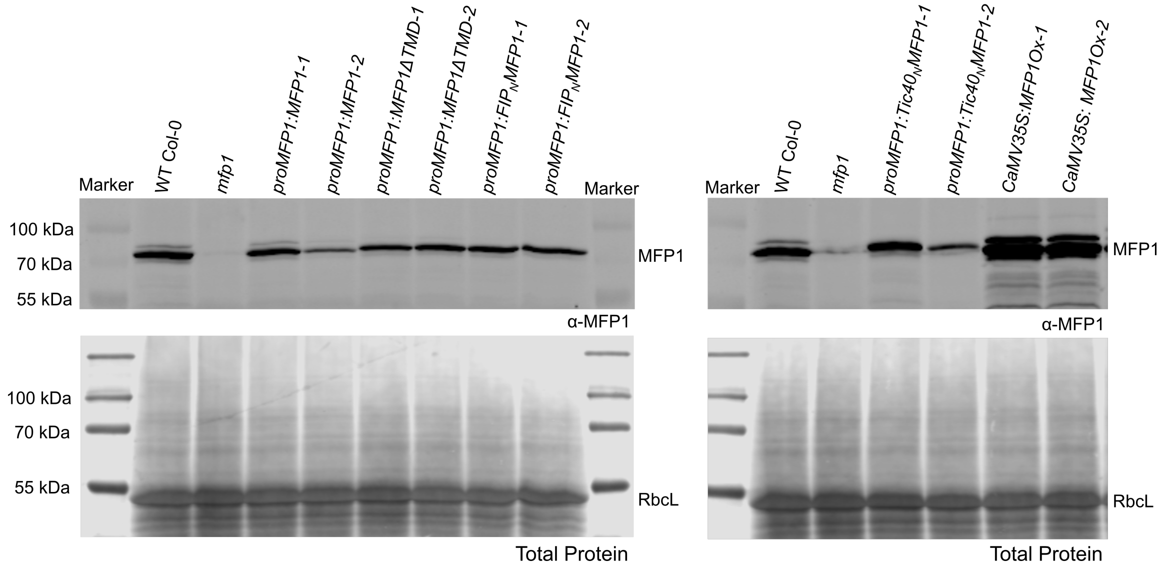

Fig. S6. Expression levels of lines expressing MFP1 variants.
Total protein extracts were loaded based on an equal fresh weight basis. Untagged MFP1 variants were detected using antibodies raised against recombinant MFP1 protein. Two independent transgenic lines are shown for each protein variant. Total proteins were stained using Revert™ 700 dye. (supplement for Fig. 3)

Figure S7

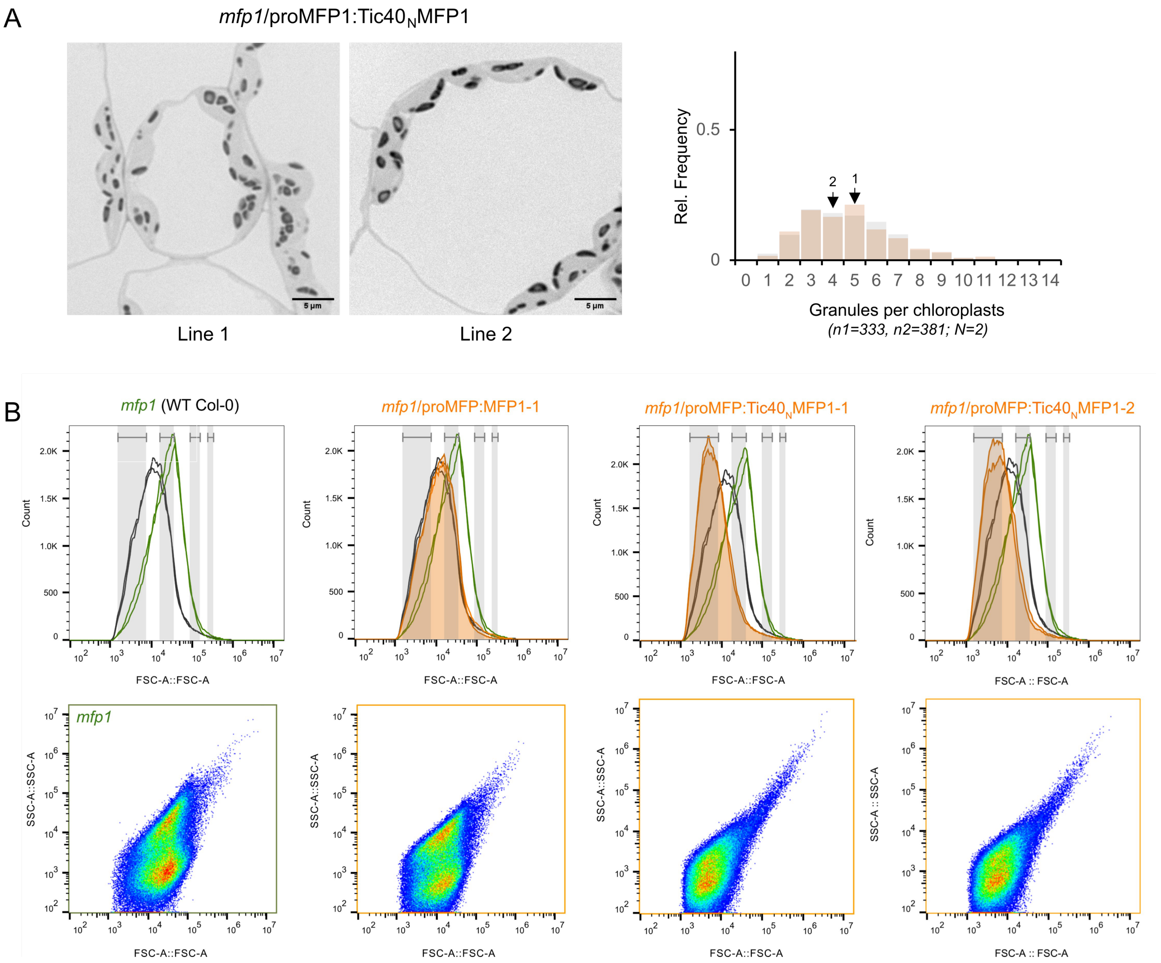

Fig. S7. Expression of envelop localized Tic40$_{N}$MFP1 affects starch granule numbers and morphology.

(A) Light microscope image of fixed leaf tissue stained with toluidine blue (*left*) and histograms of starch granule counts per chloroplast section (n) in two biological replicates (N) (*right*). Black arrow indicates medians of data from two lines. Refer to Fig. 3 for the granule counts of wild-type and *mfp1* knockout lines. Harvesting and further processing of the corresponding samples were performed in a single batch.

(B) Flow cytometry (FC) analysis of purified starch granules in two biological replicates. (*upper panels*): Histograms of the forward scatter (FSC-A) of granules purified from leaf tissues of either wild type plants (underlaid by a black line) *mfp1* knockout plants (underlaid by a green line), plants expressing wild-type MFP1 or Tic40$_{N}$MFP (orange). The first grey bar in the histogram corresponds to granule sizes ranging from 0.1-0.5µm, the second one is from 1-2 µm, the third one from 4-6 µm and the last one from 8-10µm. A shift towards smaller size is observable in the FSC of granules corresponding to Tic40$_{N}$MFP1 expressing plants. (lower panels): Two-dimensional scatter plot of forward scatter (FSC-A) against sideward scatter (SSC-A) of the respective lines. Density plots further indicate that granules purified from Tic40$_{N}$MFP1 are more spherical than those purified from the wild type or the complemented line. (supplement to Fig. 4)

Figure S8

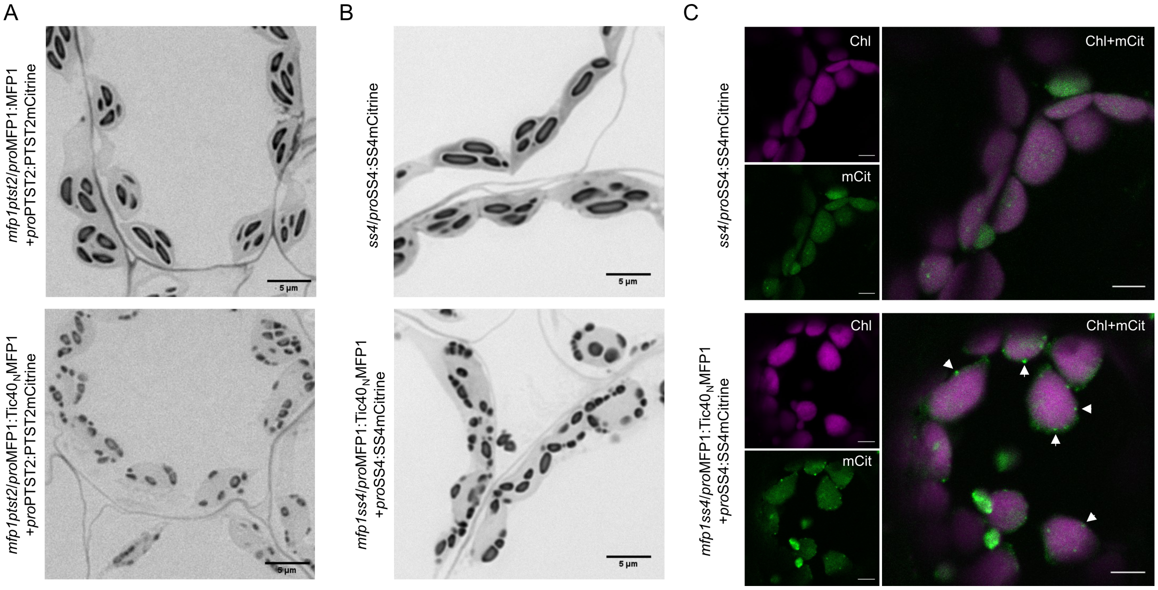

Fig. S8. Tic40$_N$MFP re-direct PTST2 and SS4 to the chloroplast envelope.

(A), (B) Light microscope image of fixed leaf tissue stained with toluidine blue showing the chloroplasts and starch granules from the indicated transgenic lines, harvested at the end of the day. The mCitrine tagged versions of PTST2 and SS4, expressed under control of their native promoters complement the starch granule phenotype of respective mutants, and their expression together with Tic40NMFP results in starch granule adjacent to chloroplast envelope.

(C) SS4-mCitrine localization in lines expressing either wild-type MFP1 or Tic40$_N$MFP, as assessed by confocal microscopy of mesophyll cell chloroplasts. The white arrows indicate some SS4 puncta at chloroplast periphery. The images were acquired early during the day, 1-2 h in the light (Supplement to Fig. 5)

Figure S9

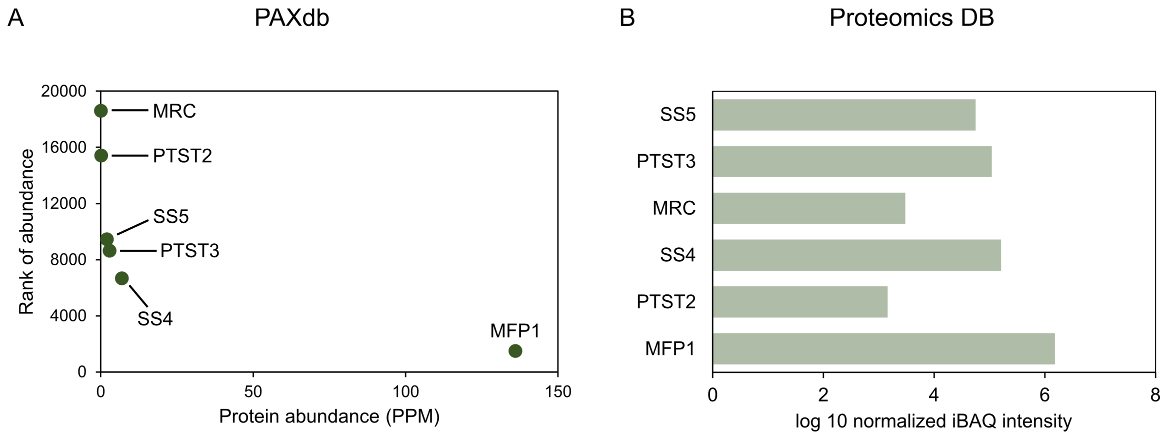

Fig. S9. Comparative abundance of starch granule initiation proteins in Arabidopsis.
(A) Plot displaying the protein abundance versus abundance ranking among 20726 identified proteins at the whole organism level (integrated). The values for selected starch granule initiation proteins were manually extracted from Pax-Db:Protein Abundance Database (1) (https://pax-db.org/), that used proteome data from Baerenfaller et al (2) and Motohashi et al (3). For comparison: the small subunit of Rubisco (AT1G67090) ranked 9 out of 20726 with abundance of 4212 PPM.
(B) Log10-transformed intensity of selected starch granule initiation proteins from leaf tissue (apex) proteome. The values were obtained from Proteomics DB (4, 5) (www.proteomicsdb.org), utilizing proteome data from Mergner et al (6). For comparison: the intensity of the small subunit of Rubisco (AT1G67090) is 8.52.
In both datasets, MFP1 emerged as the most abundant starch granule initiation protein.

References-

1. Q. Huang, D. Szklarczyk, M. Wang, M. Simonovic, C. Von Mering, PaxDb 5.0: Curated Protein Quantification Data Suggests Adaptive Proteome Changes in Yeasts. *Mol. Cell. Proteomics* **22**, 100640 (2023).
2. K. Baerenfaller, *et al.*, Genome-Scale Proteomics Reveals *Arabidopsis thaliana* Gene Models and Proteome Dynamics. *Science* **320**, 938–941 (2008).
3. R. Motohashi, A. Rödiger, B. Agne, K. Baerenfaller, S. Baginsky, Common and Specific Protein Accumulation Patterns in Different Albino/Pale-Green Mutants Reveals Regulon Organization at the Proteome Level. *Plant Physiol.* **160**, 2189–2201 (2012).
4. T. Schmidt, *et al.*, ProteomicsDB. *Nucleic Acids Res.* **46**, D1271–D1281 (2018).
5. L. Lautenbacher, *et al.*, ProteomicsDB: toward a FAIR open-source resource for life-science research. *Nucleic Acids Res.* **50**, D1541–D1552 (2022).
6. J. Mergner, *et al.*, Mass-spectrometry-based draft of the Arabidopsis proteome. *Nature* **579**, 409–414 (2020).

Legends for Movies S1 to S2

Movie S1. 3D-reconstruction of a single chloroplast, manually segmented from electron micrograph z-stack obtained after FIB-SEM of leaf tissue expressing wild type MFP1 in *mfp1* line. The chloroplast envelope, thylakoid membranes, and starch granules are colored in yellow-gray, green, and blue, respectively, in the 3D-models. (supplement to Fig. 4)

Movie S2. 3D-reconstruction of a single chloroplast, manually segmented from electron micrograph z-stack obtained after FIB-SEM of leaf tissue expressing $Tic40_N$MFP1 in *mfp1* line. The chloroplast envelope, thylakoid membranes, and starch granules are colored in yellow-gray, green, and blue, respectively, in the 3D-models. (supplement to Fig. 4)
